# *Aeromonas dhakensis*, an Increasingly Recognized Human Pathogen

**DOI:** 10.3389/fmicb.2016.00793

**Published:** 2016-05-27

**Authors:** Po-Lin Chen, Brigitte Lamy, Wen-Chien Ko

**Affiliations:** ^1^Department of Internal Medicine, National Cheng Kung University Hospital, College of Medicine, National Cheng Kung UniversityTainan, Taiwan; ^2^Center for Infection Control, National Cheng Kung University HospitalTainan, Taiwan; ^3^Laboratoire de Bactériologie-Virologie, Équipe Pathogènes Hydriques Santé Environnements, UMR 5569 HydroSciences Montpellier, Université de MontpellierMontpellier, France; ^4^Laboratoire de Bactériologie, Centre Hospitalier Régional Universitaire de MontpellierMontpellier, France

**Keywords:** *Aeromonas dhakensis*, taxonomy, identification, epidemiology, clinical infection, virulence, antimicrobial resistance

## Abstract

*Aeromonas dhakensis* was first isolated from children with diarrhea in Dhaka, Bangladesh and described in 2002. In the past decade, increasing evidence indicate this species is widely distributed in the environment and can cause a variety of infections both in human and animals, especially in coastal areas. *A. dhakensis* is often misidentified as *A. hydrophila, A. veronii*, or *A. caviae* by commercial phenotypic tests in the clinical laboratory. Correct identification relies on molecular methods. Increasingly used matrix-assisted laser desorption ionization-time of flight mass spectrometry (MALDI-TOF MS) may be able to identify *Aeromonas* specie rapidly and accurately. *A. dhakensis* has shown its potent virulence in different animal models and clinical infections. Although several virulence factors had been reported, no single mechanism is conclusive. Characteristically *A. dhakensis* is the principal species causing soft tissue infection and bacteremia, especially among patients with liver cirrhosis or malignancy. Of note, *A. dhakensis* bacteremia is more lethal than bacteremia due to other *Aeromonas* species. The role of this species in gastroenteritis remains controversial. Third generation cephalosporins and carbapenems should be used cautiously in the treatment of severe *A. dhakensis* infection due to the presence of AmpC ββ-lactamase and metallo-β-lactamase genes, and optimal regimens may be cefepime or fluoroquinolones. Studies of bacterial virulence factors and associated host responses may provide the chance to understand the heterogeneous virulence between species. The hypothesis *A. dhakensis* with varied geographic prevalence and enhanced virulence that compared to other *Aeromonas* species warrants more investigations.

## History of Taxonomy for *Aeromonas dhakensis*

*Aeromonas hydrophila* subsp. *dhakensis*, isolated from children with diarrhea in Dhaka, Bangladesh between 1993 and 1994, was first reported by Huys et al. (2002). These *A. hydrophila*-like strains initially referred to as the group BD-2 were most closely related to *A. hydrophila* DNA hybridization group (HG) 1, but examination of 152 phenotypic characteristics revealed that the group BD-2 isolates differed from the representatives of HG1 in eight biochemical properties. Martinez-Murcia et al. (2008) analyzed the strains isolated from water and skin of ornamental fish from Portugal by polyphasic approaches, including *gyrB, rpoD*, and 16S rRNA gene sequencing, DNA–DNA hybridization, matrix-assisted laser desorption ionization-time of flight mass spectrometry (MALDI-TOF MS) analysis, genotyping by random amplified polymorphic DNA, and extensive biochemical and antibiotic susceptibility tests. Martinez-Murcia et al. (2008) proposed a new species, *A. aquariorum* sp. nov.. However, 1 year later, phylogenetic analysis of three strains of *A. hydrophila* subsp. *dhakensis* using *rpoD* and *gyrB* sequencing showed that they shared the same taxon with *A. aquariorum* (Martinez-Murcia et al., 2009). Further phylogenetic study derived from 16S rRNA, *rpoD* or *gyrB* genes and a multilocus phylogenetic analysis (MLPA; with the concatenated sequences of *gyrB, rpoD, recA, dnaJ*, and *gyrA*) by Beaz-Hidalgo et al. (2013) confirmed that *A. aquariorum* and *A. hydrophila* subsp. *dhakensis* are the same taxon, and are different from the *A. hydrophila* taxon. Accordingly, formal *A. aquariorum* and *A. hydrophila* subsp. *dhakensis* have been reclassified as *A. dhakensis* sp. nov. comb nov. (Beaz-Hidalgo et al., 2013). Since then, whole genome sequence analyses unambiguously confirmed that *A. dhakensis* reached the level of species and showed that many strains have been misidentified as *A. hydrophila* (Colston et al., 2014; Beaz-Hidalgo et al., 2015).

## Characteristics of *A. dhakensis*

*Aeromonas dhakensis* strains, like other *Aeromonas* species, have typical *Aeromonas* characteristics: motile gram-negative bacilli, chemoorganotrophs with both oxidative and fermentative metabolism, cytochrome oxidase- and catalase-positivity, reduction of nitrate to nitrite without gas production, and resistance to the vibriostatic agent O/129 (Huys et al., 2002). They optimally grow after 24 h at 28°C and can also grow at 42°C on TSA medium. The phenotypic characterization of *A. dhakensis* differs from two other *A. hydrophila* subspecies (*A. hydrophila* subsp. *hydrophila* or *ranae*) by at least three biochemical properties, namely utilization of urocanic acid and L-arabinose, and acid production from L-arabinose (Huys et al., 2002, 2003).

## Species Identification

*Aeromonas dhakensis* is often clinically misidentified as *A. hydrophila* by phenotypic methods (Figueras et al., 2009). 16S rRNA sequencing has been used for more than two decades in identifying *Aeromonas* to the genus level (Martinez-Murcia et al., 1992), but the bulk 16S rRNA sequences are unreliable in identifying *Aeromonas* to the species level (Janda and Abbott, 2007). Indeed, there is on one hand a very low variability of the 16S rRNA sequence for closely related species and, on the other hand, some heterogeneity in sequences between *rrn* operons, both at an intra-genomic level (variability between copies within a given genome), an intra-species level (between strains within a given species) and an inter-species level. This hampers identification based on bulk 16S rRNA sequences (Roger et al., 2012a). Even when taking into account the multi-operon diversity of 16S rRNA, no specific signature could be identified for *A. dhakensis* and combinations of sequences could not unambiguously distinguish *A. dhakensis* from *A. caviae* (Roger et al., 2012a). Correct identification can be achieved using nucleotide sequencing of housekeeping genes, such as *rpoB, rpoD*, or *gyrB* (Martinez-Murcia et al., 2009; Wu et al., 2012b). Several studies showed that MLPA based on several housekeeping genes improves discriminative power (Martinez-Murcia et al., 2011; Martino et al., 2011; Roger et al., 2012b). For example, Martinez-Murcia et al. (2011) showed that it was achievable to clarify the genetic divergence corresponding to the intra-species or inter-species levels, based on *gyrB, rpoD, recA, dnaJ, gyrA, dnaX*, and *atpD*.

Several studies have shown that MALDI-TOF MS could efficiently identify *Aeromonas* species (Donohue et al., 2007; Lamy et al., 2011). Taxonomic identification of *A. dhakensis* by MALDI-TOF MS was firstly reported by Martinez-Murcia et al. (2008), and our work analyzing 30 clinical *A. dhakensis* isolates found that accuracy rate of MALDI-TOF MS was 96.7% (Chen et al., 2014a). However, *A. dhakensis* is not yet included in the commercial database of the MALDI-TOF system and may be reported as *A. hydrophila, A. veronii, A. caviae*, or *A. bestiarum* (Chen et al., 2014a).

## Global Distribution of *A. dhakensis*

*Aeromonas dhakensis* has been isolated from clinical specimens, animals, and environment in different countries with varying frequency and water is likely its primary habitat. *A. dhakensis* is widely distributed in tropical and subtropical areas. In environments, this species has been recovered from river water, cooling-system water pond, and fish tank water (Martinez-Murcia et al., 2008; Esteve et al., 2012). In food, *A. dhakensis* has been recovered from marine shrimps in low salinity ponds in Thailand (18% of 70 *Aeromonas* isolates, Yano et al., 2015). In addition, *A. dhakensis* was isolated from diseased eels (Esteve et al., 2012), septicemic ornemental fish (Jagoda et al., 2014), a diseased dolphin in Spain (Perez et al., 2015), chironomid egg masses in Israel (Figueras et al., 2011), and diseased fish in south Korea (Yi et al., 2013) and Mexico (Soto-Rodriguez et al., 2011). Of note, it was the predominant species, accounting for 31.4% of the *Aeromonas* isolates cultivated from farm eels in Korea (Yi et al., 2013).

## Clinical Epidemiology

With the warming climate, this species may become more prevalent in the environment along with an increased risk of exposure. Although the global epidemiology study of *A. dhakensis* is lacking, current studies show that it is prevalent in countries with hot climate, such as Bangladesh (Huys et al., 2002), Taiwan (Chen et al., 2014c; Wu et al., 2015), Australia (Aravena-Roman et al., 2011), and Malaysia (Puthucheary et al., 2012). The virulent *A. hydrophila* SSU strain recently reclassified as *A. dhakensis* SSU was originally isolated from a patient with diarrhea in Philippines (Grim et al., 2014). A case of acute gastroenteritis after returning from Egypt attributed to *A. dhakensis* occurred in Czech Republic and was concluded as an imported disease (Sedlacek et al., 2012). In countries with temperate climate, clinical infection caused by this species is rarely reported. For example, only one fatal *A. dhakensis* sepsis was reported in a cirrhotic Korean (Shin et al., 2013). Four cases of *A. dhakensis* bloodstream infection were reported in Nagasaki, Japan (Morinaga et al., 2011a). In the Roger’s study enrolling 89 and 13 clinical isolates from mainland France and its overseas territories, respectively, 5.9% (six isolates) belonged to the species *A. dhakensis*. Interestingly, four of them were recovered from a tropical or subtropical origin, e.g., either French West Indies or Reunion Island or from travelers returning from these areas (Roger et al., 2012b). However, the present information about the global distribution of *A. dhakensis* in environment and human is very likely to be underrepresented. With the increasing availability of sophisticated diagnostic tools, accurate taxonomy can improve the visibility of *A. dhakensis* in nature and patients.

## Virulence Mechanisms of *A. dhakensis*

The pathogenicity of *Aeromonas* species has been considered multifactorial. A number of virulence factors, such as secretion system, toxins, and quorum sensing system (QSS), have been discovered in the past 30 years (Tomas, 2012). The pathogenicity studies specific for *A. dhakensis* are increasing, owing to its clinical virulence and recent reclassification of taxonomy. There is converging evidence suggesting that *A. dhakensis* exhibits greater virulence compared to other *Aeromonas* species.

*Aeromonas dhakensis* (previously named *A. aquariorum*) strains have been noted to possess cytotoxic activities against human blood cell lines (Morinaga et al., 2011b) and three isolates recovered from diseased eels were virulent to healthy eels (Esteve et al., 2012). In our previous work comparing four major *Aeromonas* species associated clinical infections, *A. dhakensis* was more virulent than the other three species, i.e., *A. veronii, A. caviae*, and *A. hydrophila*, in terms of mouse fibroblast C2C12 cell line, BALB/c mouse, and *Caenorhabditis elegans* models (Chen et al., 2014d). Despite limited number of strains, Mosser et al. (2015) found that *A. dhakensis* exhibited high virulence in the *C. elegans* infection model, with unusual presentation of rapid lysis of dead body by *A. dhakensis* BVH28b. Similar events caused by the strain *A. dhakensis* AAK1 was observed in our previous work (Chen et al., 2014d). The mechanisms used by *A. dhakensis* to rapidly kill worms remain unclear (Mosser et al., 2015) and deserve to be further studied. With the progress of next generation sequencing, comparative genomics coupled with functional assays allow us to investigate the pathogen specific markers or virulence factors in the clinical or environment strains.

Over the past decades, several virulence factors and associated secretion systems have been comprehensively studied in a clinical strain, *A. dhakensis* SSU (formerly classified as *A. hydrophila* SSU). This makes *A. dhakensis* one of the most studied species for its virulence within the genus *Aeromonas* together with *A. salmonicida*. The proposed virulence factors of *A. dhakensis* SSU are summarized in **Table 1**. For example, heat-labile cytotoxic enterotoxin (Alt) and heat-stable cytotoxic enterotoxin (Ast) have been linked to clinical gastroenteritis, as both could increase cAMP levels in intestinal mucosa (Chopra and Houston, 1999). An aerolysin-related cytotoxic enterotoxin (Act) secreted by the type 2 secretion system (T2SS) is able to cause diarrheal diseases and deep wound infections in mice (Xu et al., 1998; Sha et al., 2002). A type 3 secretion system effector, AexU, with ADP-ribosyltransferase and GAP activities, plays a role in the pathogenesis of *Aeromonas* infection (Sha et al., 2007). Moreover, the type 6 secretion system (T6SS) has proved crucial in virulence through many studies in *A. dhakensis* SSU (Suarez et al., 2008, 2010b). A T6SS secreted effector, hemolysin coregulated protein (Hcp), can cause apoptosis of the host cells through activation of caspase 3 (Suarez et al., 2008), impair granulocyte activation, and cause disseminated infections in animals (Suarez et al., 2010b). Valine-glycine-repeat G (VgrG) family proteins exert cytotoxic effects by ADP-ribosylation of actin, thus prohibiting its polymerization and cytotoxicity in host cells (Suarez et al., 2010a). Recently, the exotoxin A (ExoA) has been suggested as a marked virulence factor harbored by *A. dhakensis* SSU strain (Ponnusamy et al., 2016). The ExoA-associated gene also has been identified in the genome of *A. dhakensis* BVH28b (Mosser et al., 2015). Of note, a comparative study of whole genome sequences of six *Aeromonas* strains demonstrated that *exoA* was only identified in *A. dhakensis* with particularly high virulence in *C. elegans* (Mosser et al., 2015). An *exoA*-harboring *A. hydrophila* strain was also found to be highly virulent to mice (Ponnusamy et al., 2016). However, the published data about this virulence factor are limited and the real prevalence of *exoA* in *A. dhakensis* or other *Aeromonas* species needs to be further studied.

**Table 1:** Regulatory mechanism of putative virulent factors identified in *Aeromonas dhakensis* SSU strain.

Factor	Mechanism	Effect on virulence
Alt, heat-liable enterotoxin	↑cAMP and prostaglandins in intestinal mucosa	↑
Ast, heat-stable enterotoxin	↑cAMP in intestinal mucosa	↑
Type 2 secretion system		
Act, aerolysin-related cytotoxic enterotoxin	Hemolytic, cytotoxic, and enterotoxic activities	↑
Type 3 secretion system		
AexU	GAP and ADP-ribosyltransferase, actin reorganization, apoptosis, inhibit NF-κB, inactivate Rho GTPase, ↓secretion of IL-6 and IL-8	↑
Type 6 secretion system		
Hcp, hemolysin coregulated protein	Apoptosis, necrosis of cells, impair macrophage phagocytosis, ↓pro-inflammatory cytokines, ↑suppressive cytokines	↑
Valine-glycine-repeat G	ADP-ribosylation of actin, and cytotoxicity of cells, apoptosis	↑
Quorum sensing system		
AhyRI	↑Protease production and biofilm formation	↑
LuxS	↓Secretion of T6SS-asscoiated effectors, ↓protease production and biofilm formation	↓
QseBC	↑Swimming and swarming motility ↑Production of protease and cytotoxic enterotoxin ↓Biofilm formation	↑
		↑
		↓

*↑, increase; ↓, decrease.*

*Aeromonas* species have a variety of QSSs which could modulate bacterial virulence genes (Swift et al., 1997; Kozlova et al., 2011). Consistent with this assumption, the data from Khajanchi et al. (2009) showed that a mutation of the *ahyRI* QSS of *A. dhakensis* SSU would impair the secretion of T6SS-associated effectors, protease production, and biofilm formation, if compared with the wild strain (Khajanchi et al., 2009). Another example concerns the biofilm formation that may help *Aeromonas* adhere to cell surfaces and is likely associated with gastroenteritis or wound infection (Janda and Abbott, 2010). The LuxS-based QSS inhibiting biofilm formation and decreasing virulence in a septicemic mouse model was regarded as a negative regulator of virulence in *A. dhakensis* SSU (Kozlova et al., 2008). Another QseBC QSS has been shown to both positively and negatively regulate various virulence factors of *A. dhakensis* SSU (Khajanchi et al., 2012; Kozlova et al., 2012). The decreased virulence of the Δ*qseB* mutant of *A. dhakensis* is correlated with reduced motility, and production of proteases and cytotoxic toxin. In contrast, biofilm is overexpressed if *qseB* is deleted. Moreover, ahyRI, LuxS, and QseBC systems co-regulate biofilm formation and motility of *A. dhakensis* and have complicated interactions (Kozlova et al., 2015). The balance between anhyRI and QseBC systems in *A. dhakensis* is regulated by c-di-GMP (Kozlova et al., 2011) and c-di-GMP regulation of QseBC function relies on the expression of the ahyRI system (Kozlova et al., 2012).

Although several virulence genes were reported in the SSU strain, comparative studies on the genomes of other *A. dhakensis* strains (i.e., CIP 107500, AAK1, CECT 7289, and BVH28b) showed that these virulence associated genes are not always found. Despite a body of relevant evidence argues for increased virulence (Chen et al., 2014c), virulence mechanisms remain inadequately defined. Further studies based on extensive analysis of whole genome sequences of *A. dhakensis* are needed. Accurate species and virulence factors identification may offer the chances to develop novel strategies to combat severe *A. dhakensis* infections in humans, in addition to antibiotics.

## Clinical Infections

*Aeromonas* species are often included in the list of enteropathogens and can present with invasive extra-intestinal infections, including septicemia, hepatobiliary tract infections, and soft tissue-infections. In the past, human diseases were mostly reported to be associated with three species, i.e., *A. hydrophila, A. veronii*, and *A. caviae.* However, the clinical importance given to *A. hydrophila* has been misguided because of the inaccurate species identification. With the introduction of molecular identification methods and the *A. dhakensis* description, the order of three previously predominant species has dramatically changed. *A. dhakensis*, accounting for 25.7% of identified isolates, was the second common species derived from the data of recent studies (Beaz-Hidalgo and Figueras, 2015). In a review of *Aeromonas* species distribution stratified by the isolation origin, feces was the most common origin (40.0%, 327/817), followed by wound (29.3%, 239/817) and blood (22.2%, 181/817) (Beaz-Hidalgo and Figueras, 2015). Among wound isolates, *A. dhakensis* was the predominant species (34.3%, 82/239), followed by *A. hydrophila* (27.2%, 65/239) and *A. veronii* (16.7%, 40/239).

### Skin and Soft-Tissue Infection

Skin and soft-tissue infections (SSTIs) caused by *Aeromonas* species, including cellulitis (Sanger et al., 1989; Voss et al., 1992), abscesses (Voss et al., 1992; Gold and Salit, 1993), necrotizing fasciitis (Minnaganti et al., 2000), and myonecrosis (Voss et al., 1992; Vukmir, 1992; Moses et al., 1995), are common clinical manifestations. Severe SSTIs have been reported to be mainly associated with *A. hydrophila* (Chao et al., 2013). However, the incidence of SSTIs caused by *A. dhakensis* may have been underestimated, as this species may be reported as *A. hydrophila* based on phenotypic tests (Lamy et al., 2010; Aravena-Roman et al., 2011; Chao et al., 2013) or prior to the species description of *A. dhakensis*. Given the recognized importance of *A. dhakensis* in diseases, epidemiologic data before the taxonomic description of *A. dhakensis* should be considered as outdated, at least for the prevalence of *A. hydrophila*. In our research, *A. dhakensis* was the most prevalent (46.3%) among 80 *Aeromonas* wound isolates in southern Taiwan (Chen et al., 2014c). Among the 11 patients with monomicrobial *Aeromonas* wound infections, seven were infected by *A. dhakensis* and three evolved into necrotizing fasciitis requiring surgical fasciotomy (Chen et al., 2014c).

### Bacteremia

*Aeromonas* bacteremia usually occurs in patients with underlying illnesses, such as malignancy, liver cirrhosis, diabetes mellitus, or receipt of immunosuppressant therapy (Tang et al., 2014). Previous studies indicated that *A. hydrophila, A. caviae*, and *A. veronii*, accounted for the majority of *Aeromonas* bacteremia (Chuang et al., 2011; Tang et al., 2014). Our recent work revealed that among 153 episodes of monomicrobial *Aeromonas* bacteremia, *A. veronii* (50 isolates, 32.7%), *A. dhakensis* (48, 31.4%), *A. caviae* (43, 28.1%), and *A. hydrophila* (10, 6.5%) were the principal causative species (Wu et al., 2015). The origins of *A. dhakensis* bacteremia were mainly unrecognized (47.9%), spontaneous bacterial peritonitis (16.7%), biliary tract infection (10.4%), and skin and soft-tissue infection (10.4%). A substantial proportion of *A. dhakensis* bacteremia was community-onset (70.8%) and occurred in cirrhotic patients (62.5%). Of note, *A. dhakensis* was linked to the highest 14-day sepsis-related mortality rate (25.5%) compared with *A. veronii* (14.0%) and *A. caviae* (4.7%). Because of the distribution of *A. dhakensis* in the environment and aquatic creatures (Martinez-Murcia et al., 2008; Aravena-Roman et al., 2011), oral ingestion or abraded wounds can serve as the portals of entry of *A. dhakensis* infection.

### Gastroenteritis

*Aeromonas dhakensis* was first isolated from children with diarrhea in Dhaka, Bangladesh (Huys et al., 2002) and the *A. dhakensis* SSU was recovered from a diarrheal patient during a cholera-like outbreak (Grim et al., 2014). In a review of *Aeromonas* species identified by molecular methods among clinical samples, 43.6% (82 of 188) of *A. dhakensis* were isolated from feces (Beaz-Hidalgo and Figueras, 2015). In Taiwan, Wu et al. (2012b) reported a cirrhotic patient with bloody diarrhea, in whom *A. dhakensis* identified using *rpoD* and *rpoB* sequencing was concurrently isolated from blood and feces. Significant *A. dhakensis*-induced cytotoxicity to intestinal epithelial cells was demonstrated *in vitro*. Among 11 Taiwanese adults with *Aeromonas*-associated diarrhea, only one *A. dhakensis* isolate was obtained from an 83-year-old female with an uneventful hospital course (Chen et al., 2014b). Most of the fecal isolates of *A. dhakensis* originated from Asia or tropical areas, suggestive of geographic variation of prevalence.

## Antimicrobial Resistance and Therapy

*Aeromonas dhakensis* is susceptible to cefepime, aminoglycosides, fluoroquinolones, and tetracyclines (Chen et al., 2014c; Wu et al., 2015). Although antimicrobial resistance of broad-spectrum cephalosporins or carbapenems was not common in *A. dhakensis* isolates (Chen et al., 2014a,c; Wu et al., 2015), this species intrinsically harbors class B (metallo-β-lactamases, MBLs; CphA), C (AmpC cephalosporinase; AQU-1), and D β-lactamases (penicillinases; Wu et al., 2012a, 2013). CphA, a MBL found in *A. dhakensis, A. veronii*, and *A. hydrophila* (Wu et al., 2012a), has a specific substrate profile of hydrolyzing carbapenems but not penicillins or cephalosporins, if compared with other MBLs (Segatore et al., 1993). However, CphA carbapenemase production is not easily detected by the conventional susceptibility test, unless a large inoculum or additional tests (e.g., modified Hodge test, ertapenem MIC or Carba NP test) are applied (Rossolini et al., 1995; Wu et al., 2012a; Sinclair et al., 2016). Carbapenem therapy for *cphA-*carrying *Aeromonas* infections remains controversial, because of the clinical concern that carbapenem monotherapy may fail in treating MBL-producing aeromonads infections with a high bacterial burden, such as peritonitis/abdominal sepsis or soft tissue infections (Wu et al., 2012a). Moreover, breakthrough bacteremia due to *A. dhakensis* during ertapenem therapy has been reported (Wu et al., 2012a). Therefore, performing the susceptibility tests with a large inoculum and/or with additional tests before carbapenem therapy is advised for severe *A. dhakensis* infections. Optimal antimicrobial choices for *A. dhakensis* infections may include a fluoroquinolone or cefepime before the drug susceptibility is conclusive.

AmpC β-lactamases can hydrolyze cephamycins and third generation cephalosporins, and are resistant to β-lactamase inhibitors, such as clavulanic acid, tazobactam, and sulbactam (Bush et al., 1995). The distribution of the AmpC β-lactamase among *Aeromonas* strains is species-specific, i.e., *bla_AQU-1_, bla_MOX_*, or *bla_CepH_* was present in *A. dhakensis, A. caviae*, or *A. hydrophila*, respectively (Wu et al., 2015). These findings suggest that clinical use of broad-spectrum cephalosporins, except cefepime, for *A. dhakensis* infections, should be used with caution before supporting antimicrobial susceptibility testing data are available.

## Conclusion

*Aeromonas dhakensis* is a demonstrative example that accurate taxonomy can improve our knowledge about epidemiological distribution and virulence potential of human pathogens. At present time, accurate identification of *A. dhakensis* is encouraged because of its virulence and potential antimicrobial resistance. Consequently, improved recognition of *A. dhakensis* will lead to revise our considerations on actual pathogenic potential and clinical impact of *A. hydrophila* infection. More researches to re-estimate clinical role of *A. hydrophila* in human infection, to disclose disease burden of *A. dhakensis*, and to understand how this enhanced virulent species could emerge from the *Aeromonas* complex, are warranted.

## Author Contributions

Composed the article: PLC; drafted the paper: PLC; critically commented and revised the manuscript: WCK and BL. All authors read and approved the final manuscript.

## Conflict of Interest Statement

The authors declare that the research was conducted in the absence of any commercial or financial relationships that could be construed as a potential conflict of interest.
